# Epithelial Notch signaling is a limiting step for pancreatic carcinogenesis

**DOI:** 10.1186/1471-2407-14-862

**Published:** 2014-11-22

**Authors:** Marsha M Thomas, Yaqing Zhang, Esha Mathew, Kevin T Kane, Ivan Maillard, Marina Pasca di Magliano

**Affiliations:** Department of Surgery, 1500 E Medical Center Drive, Ann Arbor, Michigan 48109-5936 USA; Cell and Molecular Biology Program, University of Michigan, 2966 Taubman Medical Library, Ann Arbor, MI 48109-0619 USA; Life Science Institute, University of Michigan, 210 Washtenaw Avenue, Ann Arbor, MI 48109-2216 USA; Department of Cell and Developmental Biology, University of Michigan, 3059 A. Alfred Taubman Biomedical Science Research Building, 109 Zina Pitcher Place, Ann Arbor, MI 48109-2200 USA

**Keywords:** Pancreatic cancer, Notch, DNMAML, Mastermind-like, Epithelium, Genetically engineered mouse model

## Abstract

**Background:**

Pancreatic cancer is one of the deadliest human malignancies, with few therapeutic options. Re-activation of embryonic signaling pathways is commonly in human pancreatic cancer and provided rationale to explore inhibition of these pathways therapeutically. Notch signaling is important during pancreatic development, and it is re-activated in pancreatic cancer. The functional role of Notch signaling during pancreatic carcinogenesis has been previously characterized using both genetic and drug-based approaches. However, contrasting findings were reported based on the study design. In fact, Notch signaling has been proposed to act as tumor-promoter or tumor-suppressor. Given the availability of Notch inhibitors in the clinic, understanding how this signaling pathway contributes to pancreatic carcinogenesis has important therapeutic implications. Here, we interrogated the role of Notch signaling specifically in the epithelial compartment of the pancreas, in the context of a genetically engineered mouse model of pancreatic cancer.

**Methods:**

To inhibit Notch signaling in the pancreas epithelium, we crossed a mouse model of pancreatic cancer based on pancreas-specific expression of mutant Kras with a transgenic mouse that conditionally expresses a dominant negative form of the Mastermind-like 1 gene. MAML is an essential co-activator of the canonical Notch signaling-mediated transcription. *DNMAML* encodes a truncated MAML protein that represses all canonical Notch mediated transcription in a cell autonomous manner, independent of which Notch receptor is activated. As a result, in mice co-expressing mutant *Kras* and *DNMAML*, Notch signaling is inhibited specifically in the epithelium upon Cre-mediated recombination. We explored the effect of epithelial-specific DNMAML expression on Kras-driven carcinogenesis both during normal aging and following the induction of acute pancreatitis.

**Results:**

We find that DNMAML expression efficiently inhibits epithelial Notch signaling and delays PanIN formation. However, over time, loss of Notch inhibition allows PanIN formation and progression.

**Conclusions:**

Epithelial-specific Notch signaling is important for PanIN initiation. Our findings indicate that PanIN formation can only occur upon loss of epithelial Notch inhibition, thus supporting an essential role of this signaling pathway during pancreatic carcinogenesis.

**Electronic supplementary material:**

The online version of this article (doi:10.1186/1471-2407-14-862) contains supplementary material, which is available to authorized users.

## Background

Pancreatic ductal adenocarcinoma (PDA), the most common form of pancreatic cancer, is a highly aggressive malignant disease with a very poor prognosis. It is the fourth most common cause of cancer-related mortality across the US and other developed countries independent of race or sex, as the number of new cases and disease-related deaths are roughly equal [1, 2]. There is a dire need for new therapeutic options for this disease to increase the dismal 5-year survival, which is currently less than 5%.

PDA develops through a series of non-invasive precursor lesions, the most common of which are pancreatic intraepithelial neoplasia or PanINs [3]. Oncogenic mutations in KRAS are widespread in human PanINs and detected in in over 90% of human PDA [3–6]. In addition, PanIN progression is marked by aberrant activation of embryonic signaling pathways, like Hedgehog, Wnt, and Notch [3–5, 7–9]. Expression of oncogenic Kras in genetically engineered mice recapitulates the step-wise progression of the human disease, and gives rise to one of the most commonly used mouse models of pancreatic cancer, the KC mouse [4].

Notch signaling induces the expansion and transformation of an undifferentiated precursor population during pancreatic cancer progression [9–13]. Extensive analysis of human pancreatic cancer revealed activation of Notch signaling both in PanINs and in PDA [4, 9]. Notch has been shown to be important for stimulating proliferation in transformed cells [10]. A recent study used a γ-secretase inhibitor (GSI) to block Notch signaling in KPC mice [10], resulting in reduced incidence of PanIN lesions and decreased proliferation of PanINs. These studies and others imply Notch signaling may regulate proliferation of transformed acinar-ducal metaplasia (ADM) structures and PanINs [10]. Nevertheless, GSIs could block other functions in the cell other than Notch signaling, since γ-secretase targets other proteins in addition to Notch components [14]. Furthermore, GSIs systemically blocks all Notch activity independent of cell types, making it difficult to tease apart the details of Notch signaling in pancreatic cancer cells versus effects on the stroma [14].

Several studies have used genetic approaches to ablate individual Notch receptors and study the effect on pancreatic carcinogenesis. Interestingly, these experiments resulted in contrasting findings depending on the specific Notch receptor being targeted, with either tumor suppressive or tumor promoting effects being observed [10, 12, 13, 15–17]. Thus, a study addressing complete ablation of Notch signaling in the pancreatic epithelium in KC mice was so far missing.

Here, we report the use of a dominant negative form of MAML1 (DNMAML), a critical regulator of canonical Notch activity, to address the requirement of Notch signaling within the tumor epithelium during pancreatic tumorigenesis [18].

## Methods

### Mouse strains

Animals were housed in pathogen-free conditions and maintained in facilities of the University of Michigan Comprehensive Cancer Center and this study was included in our animal use protocol, approved by the University of Michigan University Committee on Use and Care of Animals (UCUCA). P48-Cre (Ptf1a-Cre) [19] (provided by Christopher V. Wright, Vanderbilt University, Nashville, Tennessee, USA) and LSL-*Kras*^G12D^[20] (provided by David Tuveson, Cambridge Research Institute, Cambridge, United Kingdom) were crossed to generate KC mice, as previously described [4, 20]. *ROSA*^*DNMAML/+*^ mice [21, 22] were crossed with KC mice to generate KC;DNMAML mice. Wild type mice and animals having a combination of Kras allele and/or DNMAML, but not the Cre allele were used as controls. Allele-specific PCR on mouse tail DNA, was used to verify the presence of each allele. DNMAML induction was monitored through GFP expression in pancreas epithelium. 5 animals per cohort (≥5 mice/cohort) were aged 15 and 26 weeks before euthanasia. Tissues were collected for histopathological analysis.

### Genotyping

Tail DNA was extracted using hot sodium hydroxide and tris (HotSHOT) DNA extraction protocol, as previously described [23]. The primers used for p48-Cre, *Kras*^*G12D*^, and *ROSA*^*DNMAML/+*^ were as follows: *p48-Cre*, 5’-catgcttcatcgtcggtcc- 3’ (forward) and 5’-gatcatcagctacaccagag-3’ (reverse); *Kras*^*G12D*^, 5’-agctagccaccatgagtaagtctgca-3’ (forward) and 5’-cctttacaagcgccgcagactgtaga-3’ (reverse); Rosa^DNMAML-GFP/+^, 5’-aaagtcgctctgagttgttat-3’ (Rosa1), 5’-gcgaagagtttgtcctcaacc-3’ (Rosa2), and 5’-ggagcgggagaaatggatatg-3’ (Rosa3). PCR cycling conditions were as follows: p48-Cre, Kras^G12D^, 95°C for 3 min, 95°C for 30 s, 60°C for 30 s, and 72°C for 45 s for 34 cycles, followed by 72°C for 5 min; Rosa^DNMAML-GFP/+^, 95°C for 3 min, 95°C for 30 s, 59°C for 30 s, and 72°C for 1 min for 34 cycles, followed by 72°C for 5 min. Amplified PCR products were run on 2% agarose gels with molecular weight markers. PCR products were visualized under Alpha Innotech UV transilluminator.

### Induction of acute pancreatitis

Animals were administered caerulein (Sigma-Aldrich) by intraperitoneal injections in two series of 8 hourly at a concentration of 75 μg/kg over a 48 hour period, as previously described [24]. Age-matched controls were injected in parallel with experimental mice.

### Immunohistochemistry and immunofluorescence

Pancreatic tissues from experimental and control mice were dissected and fixed overnight in 10% neutral-buffered formalin (Fisher Scientific) embedded in paraffin and sectioned (4–5 μm). The University of Michigan Cancer Center Histopathology Core performed all embedding and sectioning. Paraffin-embedded tissue sections were processed using 2 × xylene for 5 min, 2 × 100% ethanol for 5 min, 2 × 95% ethanol for 2 min, and rinsed under running deionized water for 5 min. Antigen retrieval was performed using citrate buffer (BioGeneX) in the microwave and cooled. Blocking of endogenous peroxidase activity was achieved using 3% hydrogen peroxide for 10 min, and then sections were blocked using 5% albumin from bovine serum (BSA; Sigma-Aldrich) for 30 min. Hematoxylin/Eosin (H&E), Periodic Acid Staining (PAS), Gomori trichrome, and immunohistochemistry staining was performed as previously described [5]. For a list of antibodies used, see Additional file 1: Table S1. Images were taken with an Olympus BX-51 microscope, Olympus DP71 digital camera, and DP Controller software.

For immunofluorescence, secondary-antibodies labeled with Alexa Fluor 488 (Life Technologies). Cell nuclei were counterstained with 4’,6-diamidino-2-phenylindole (DAPI; Invitrogen). The immunofluorescent images were acquired using an Olympus IX-71 confocal microscope and FluroView FV500/IX software.

### Hes1 staining

Paraffin-embedded tissue sections were processed as described. *Hes1* antibody (a gift from Ben Stanger, University of Pennsylvania, Philadelphia, PA, USA) at a 1:1500 dilution was amplified using Tyramide Signal Amplification system (PerkinElmer). Alexa Fluor 5. was used to visualize staining by immunofluorescence (Life Technologies).

### Quantitative real time PCR

Tissue for RNA extraction was stabilized through overnight incubation in RNA*later*-ICE (Ambion) at −20°C, then isolated using RNeasy Mini Kit (QIAGEN) according to manufacturer’s instructions. Reverse transcription reactions were conducted using a High-Capacity cDNA Reverse Transcription Kit (Applied Biosystems). Samples for quantitative RT-PCR were prepared with 1× Power SYBR Green PCR Master Mix (Applied Biosystems) and various primers (Additional file 1: Table S2). All primers were optimized for amplification under reaction conditions as follows: 95°C for 10 min, followed by 40 cycles of 95°C for 15 s and 60°C for 1 min. Melting curve analysis was performed for all samples after completion of the amplification protocol. *Cyclophilin* and *Gapdh* were used as the reference gene expression controls. *Hes1* primers were acquired from Applied Biosystems; Mouse *Hes1* (Mm01342805_m1), Mouse *Gapdh* (Mm99999915_g1). Amplification was preformed using the StepOnePlus System (Applied Biosystems), and experiments were performed in triplicates. The results were calculated following the 2ΔC_*p*_ method using the StepOne Software (Applied Biosystems). A two-tailed unpaired t test was used for statistical analysis.

### Histopathological analysis

The histopathological analysis was performed as previously described [25]. In brief, 5 randomly selected, non-overlapping high-power images (20× objective) were taken for each slide. A minimum of 50 total acinar or ductal clusters was counted from at least three independent animals for each group. Each cluster counted was classified as acinar, ADM, PanIN1A, PanIN1B, PanIN2, and PanIN3 based on the classification consensus [26]. Prism software was used to perform statistical tests and assess statistical significance. (GraphPad; Mac version 6.0). A *P* values were calculated according to multiple t test and were considered significant when less than 0.05 and highly significant when less than 0.01.

## Results

### Epithelial-specific inhibition of Notch signaling

**Figure 1:**
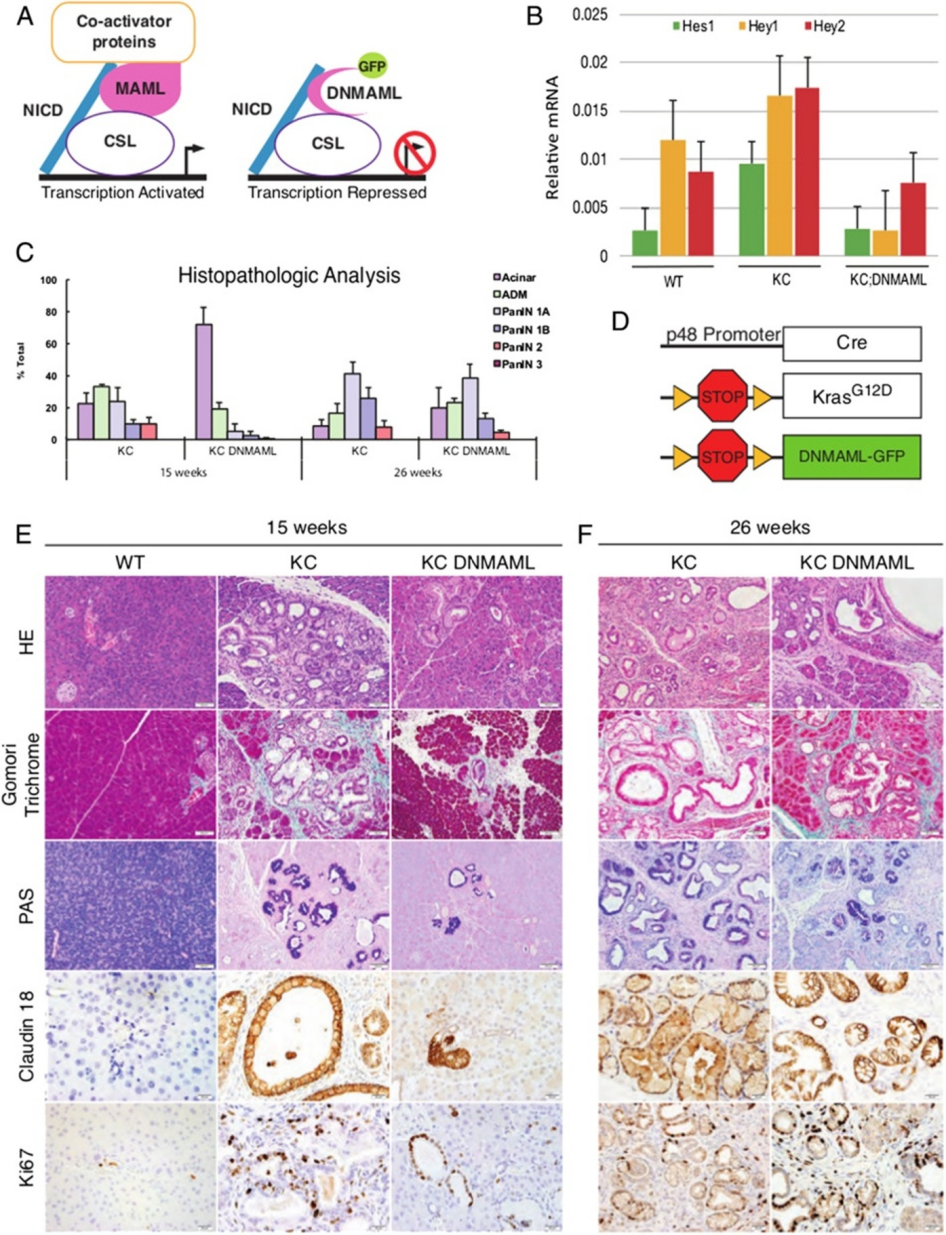
**Epithelial-specific inhibition of Notch signaling in DNMAML model. (A)** Scheme of DMAML-mediated inhibition of Notch signaling. The Notch intracellular domain (NICD; blue) forms a complex with suppressor of hairless and lag-1 (CSL; purple lined), Mastermind-like1 (MAML; pink), and co-activators (yellow lined), to activate transcription of Notch target genes. The DNMAML-GFP fusion protein blocks formation of the activation complex, thus preventing transcription of the target genes. **(B)** Pancreata from 15 week-old wild type (WT), *p48Cre;LSL-* ^*KrasG12D*^ (KC), and *p48Cre;LSL-* ^*KrasG12D*^ mice (KC;DNMAML) were analyzed for the expression of common Notch target genes, *Hes1* (green column), Hey1 (yellow column) and Hey2 (red column) by qRT-qPCR. The expression levels were normalized to *Gapdh.* Data represent mean ± SEM. **(C)** Quantification of pancreatic intraepithelial neoplasia (PanIN) of age-matched KC and KC;DNMAML mice (*n* = 3-5 mice/genotype). Color-coding: acini (dark purple), acinar-ductal metaplasia (ADM; green), PanIN1A (light blue), PanIN2B (blue), PanIN2 (red), PanIN3 (dark red). Data represent mean ± SEM. The statistical difference was determined by two-tailed Student t-test. *p < 0.05, ***p < 0.0001. **(D)** Genetic makeup of KC;DNMAML mice. **(E)** Analysis of 15-week old mice. H&E staining (20×); collagen deposition (Gomori Trichrome; 20×); mucin accumulation (Periodic Acid Staining; PAS; 20×); a PanIN-specific marker (Claudin18; 40×), and proliferation (Ki67; 40×). **(F)** Analysis of 26-week old mice.

To block Notch signaling during pancreatic carcinogenesis, we crossed a mouse model of pancreatic cancer, based on pancreas-specific expression of mutant Kras (p48-Cre; LSL-Kras^G12D^; hereby referred to as KC) with a transgene expressing a Dominant-Negative form of *Mastermind-like1* (*DNMAML*), which encodes for a truncated form of MAML1 (aa 13–74) fused to GFP, downstream of a LoxP-flanked stop cassette [5, 6, 21] (Figure 1D). The DNMAML-GFP fusion product is a potent inhibitor of Notch 1–4 signaling *in vivo* and *in vitro* and interferes with the NICD-CSL/RBPJ-MAML complex formation, which is essential for transcriptional activation of Notch target genes in a cell autonomous manner [18, 27] (Figure 1A). The DNMAML-GFP allows tracking recombination events in individual cells by taking advantage of the GFP fusion protein [21].

To verify the ability of DNMAML to inhibit Notch signaling in the pancreas, we harvested pancreata from 15 week old wild type, KC, and KC;DNMAML mice and analyzed the expression of Notch target genes in whole-tissue mRNA (5 mice/cohort). Expression of *Hes1*, *Hey1*, and *Hey2*, common targets of Notch signaling was assessed by quantitative real time polymerase chain reaction (RT-qPCR) [28–30] (Figure 1B). We observed elevated levels of *Hes1*, *Hey1*, and *Hey2* in KC mice compared to WT control samples, in accordance with previously published data showing Notch signaling upregulation in PanINs and PDA [4, 9, 10]. Importantly, the Notch target genes were downregulated in KC;DNMAML mice compared to KC. Thus, DNMAML expression successfully inhibited Notch signaling in the pancreas *in vivo*.

### Epithelial Notch signaling is important for PanIN initiation

In order to determine the effects of Notch inhibition in the epithelial compartment during pancreatic carcinogenesis, we harvested tissue from age-matched KC and KC-DNMAML mice at 15 (n = 5) and 26 (n = 28) weeks of age and examined tissue histology for neoplastic progression. In 15 week-old samples we observed a significant reduction in the number of PanINs in KC;DNMAML mice compared to KC mice (Figure 1E). Thus, inhibition of Notch signaling delayed or blocked PanIN formation. Analysis of those PanINs that were present in KC;DNMAML mice did not reveal any significant histological difference compared to PanINs in KC mice. Then, we examined the pancreata of KC;DNMAML and KC mice dissected at 26 weeks of age (Figure 1F). At this time point, there was no significant difference in the number of PanINs between KC and KC;DNMAML mice. There results were consistent with Notch inhibition being insufficient to block PanIN formation, or with loss of Notch inhibition through a negative selection process. We performed histological and molecular analysis of PanINs in KC and KC;DNMAML tissues. PanIN lesions from both genotypes expressed typical markers such as Claudin18 and showed intracellular mucin staining (PAS staining), in addition to collagen deposition (Gomori Trichrome staining). To compare the proliferation index among the two genotypes, we performed Ki67 staining [31]. At 15 weeks and 26 weeks, we observed elevated Ki67 staining in both the tumor epithelium and stromal compartment, independent of genotype (Figure 1E,F). We then analyzed apoptosis, by cleaved Caspase3 staining and did not observe any difference in the two sets of mice (data not shown). Thus, once PanINs had formed, they had the expected proliferation and cell survival rate.

Histopathological analysis of de-identified slides confirmed our initial observations. Namely, KC;DNMAML samples had more acinar clusters compared to KC samples (80% and 30% respectively) and less ADM (20% and 35%, respectively) at 15 weeks. KC pancreata displayed a greater degree of disease progression with a higher proportion of PanIN1A (20%), PanIN1B (8%), and PanIN2 (1%) compared to age-matched KC;DNMAML mice (Figure 1C). At 26 weeks, we observed significantly fewer PanINs and more acini in KC;DNMAML mice compared to KC. Moreover, KC;DNMAML had lower-grade PanINs than KC samples, reflecting the delay in lesion onset (Figure 1C). Thus, PanINs were delayed, but their formation was not blocked, upon inhibition of epithelial Notch signaling. This finding might be explained by progressive loss of Notch-dependency (possibly due to compensation by other signaling pathways) or by loss of Notch inhibition due to competitive disadvantage –thus elimination- of DNMAML expressing cells.

**Figure 2:**
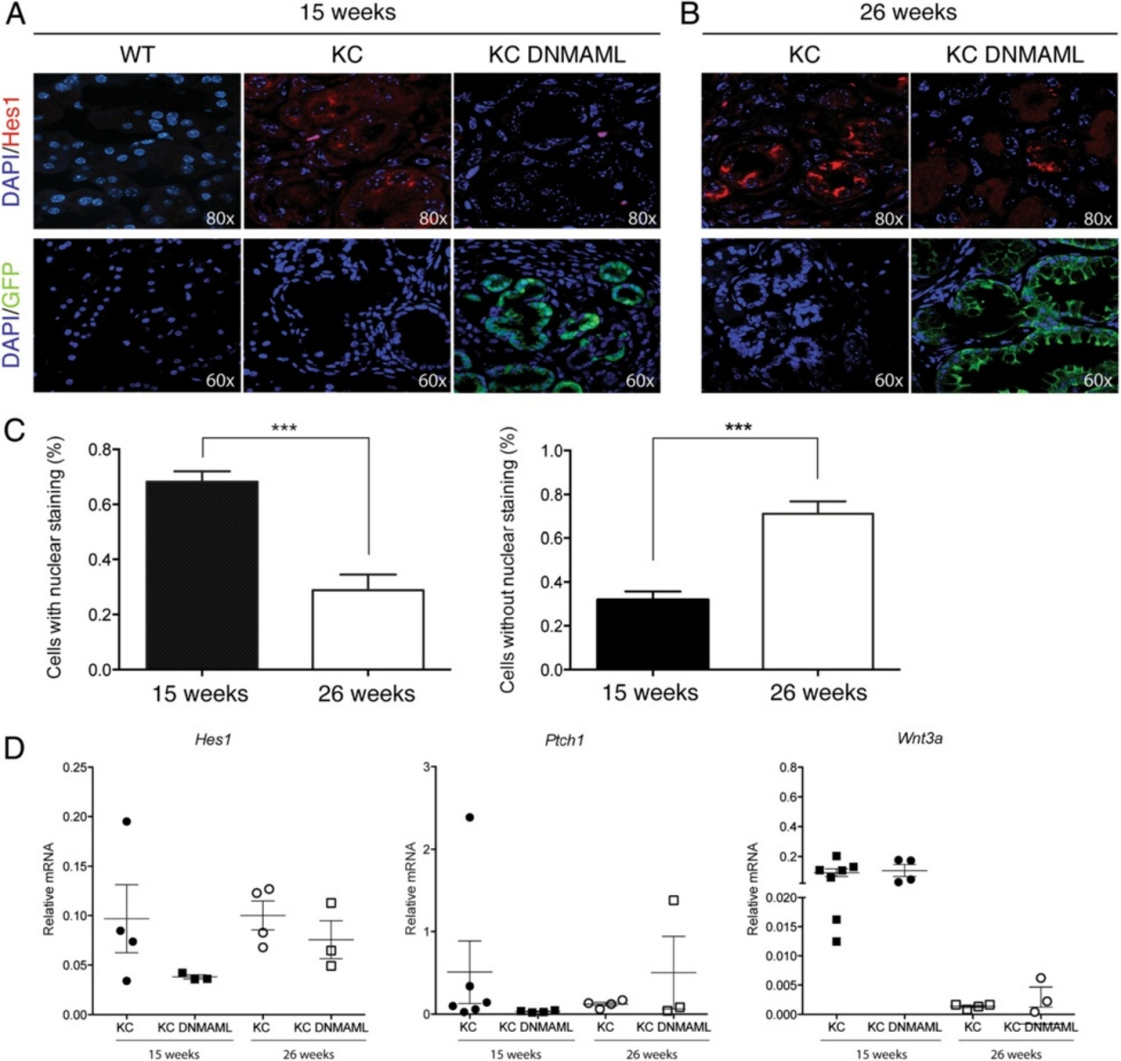
**Progressive loss of Notch inhibition.** Pancreata were harvested at the indicated time points from 15-week **(A)** and 26-week old **(B)** wild type (WT), *p48Cre;LSL-Kras* ^*G12D*^ (KC), and *p48Cre;LSL-Kras* ^*G12D*^ *; Rosa* ^*DNMAML-GFP/+*^ (KC;DNMAML) mice. Immunofluorescence images represent Notch activity (Hes1; red; 80×) and DNMAML expression (GFP; green; 60×). To identify the nucleus, all sections were stained with DAPI. **(C)** Quantification of DNMAML expression (GFP) within the nucleus or within the cytoplasm at 15 weeks and 26 weeks. Data represents mean ± SEM. ***, *P* < 0.001. **(D)** RT-qPCR analysis of Hedgehog signaling component, Patched 1(*Ptch1*) and Wnt ligand, Wnt3a (*Wnt3a*) in KC and KC;DNMAML mice at the indicated time points. Each point represents 1 mouse. Data represents mean ± SEM. *Gapdh* and *Cyclophilin* were used as reference genes.

In order to determine whether DNMAML expression had been lost over time, or whether Notch signaling was still inhibited in the epithelial compartment, we analyzed Hes1 and GFP protein localization by immunofluorescence (Figure 2). At 15 weeks, Hes1 was upregulated in KC samples but not in KC-DNMAML tissues as predicted. GFP staining overlapped with DAPI nuclear staining, suggesting that DNMAML was localized in the nuclear compartment (Figure 2A and C). At 26 weeks, Hes1 appeared further upregulated in KC samples, and we also observed Hes1 expression in the PanINs of KC-DNMAML tissues, suggesting increased Notch activity (Figure 2B and C). Moreover, we observed loss of nuclear GFP, and concurrent appearance of cytoplasmic or membrane GFP. While we can’t fully explain the mechanism underlying the latter finding, we nevertheless were able to conclude that the DNMAML-mediated inhibition of Notch signaling was lost over time, either by loss of DNMAML expression or DNMAML-expressing cells, or by inappropriate subcellular localization of the DNMAML-GFP fusion protein. Thus, the loss of phenotype in KC;DNMAML mice over time coincided with re-activation of Notch signaling. These data thus support an essential requirement for epithelial Nocth signaling during PanIN formation.

To further our analysis of KC;DNMAML mice, we investigated whether inhibition of Notch signaling affected the activation of other embryonic signaling pathways that are upregulated in PanIN formation and important for their progression, such as Hedgehog and Wnt [5, 8, 32–35]. Thus, we harvested tissue from 15 and 26 week old KC;DNMAML and age-matched KC mice and extracted mRNA to analyze Hedgehog and Wnt associated gene expression, such as Patched-1 (*Ptch1*), a Hedgehog pathway component and target gene, and Wnt3a (*Wnt3a*), a Wnt ligand [5, 8] (Figure 2D).

In the same set of experiments, we measured Hes1 expression by qPCR, to determine the degree of Notch inhibition in the individual samples (Figure 2D). At 15 weeks, we observed a trend towards a decrease in *HES1* in KC-DNMAML samples (n ≥3) when compared to KC samples, as predicted. Our data did not reach statistical significance, possibly due to the heterogeneous nature of whole tissue samples. At 26 weeks, there was no difference in *HES1* expression between KC and KC-DNMAML samples, corroborating our immunostaining-based results described above. At neither time point did we observe a difference in *Ptch-1* and *Wnt3a* expression. Thus, inhibiting Notch signaling had no effect on Hedgehog and Wnt activity.

**Figure 3:**
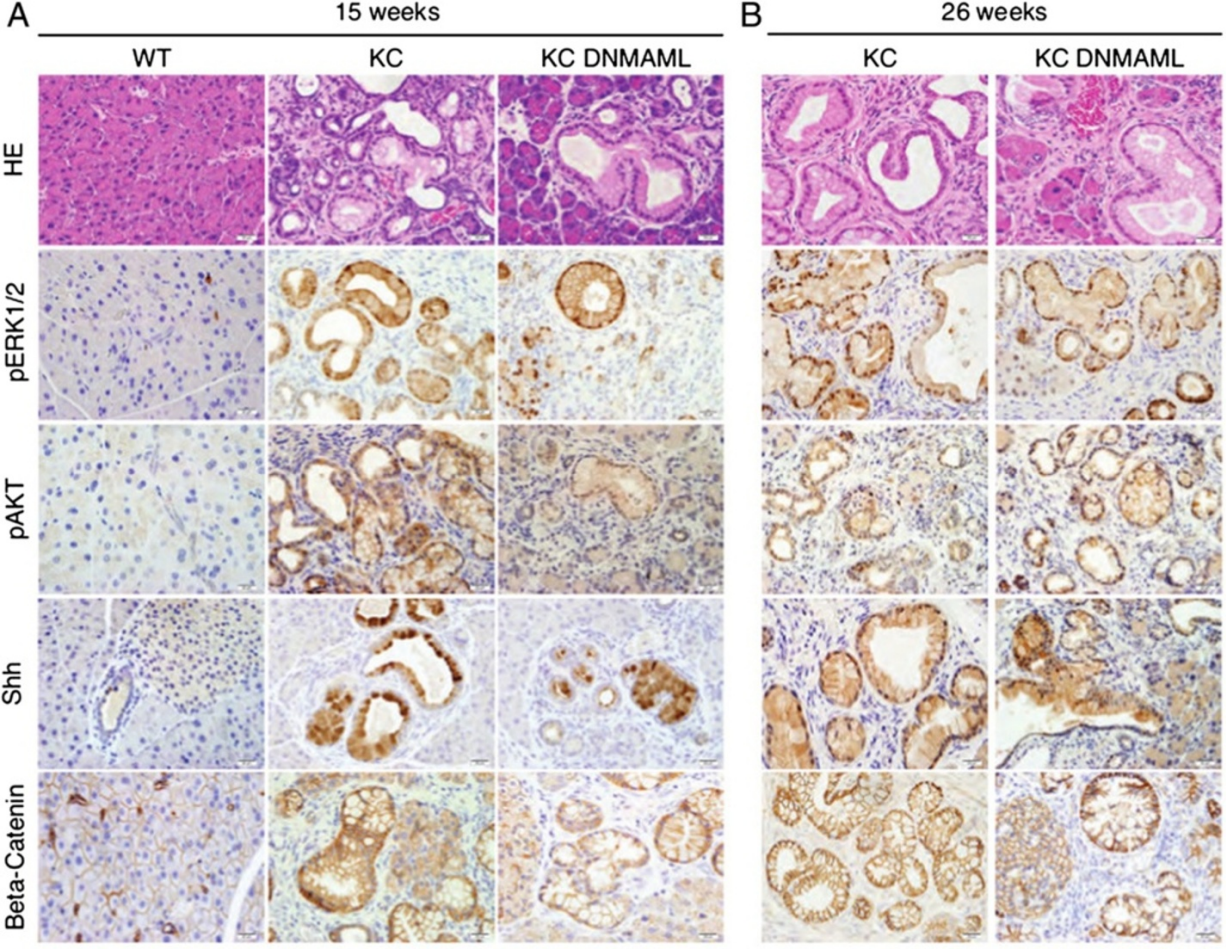
**Histological analyses of Kras, Hedgehog, and Wnt signaling components. (A)** Pancreata was harvested and analyzed from 15-week old wild type (WT), *p48Cre;LSL-Kras* ^*G12D*^ (KC), and *p48Cre;LSL-Kras* ^*G12D*^ *; Rosa* ^*DNMAML-GFP/+*^ (KC;DNMAML) mice. H&E staining at a high magnification (40×) compare the pancreatic histology in WT, KC, KC;DNMAML mice. Tissues were stained for downstream targets of Kras signaling (p-AKT and p-ERK), Hedgehog ligand (Shh), and downstream Wnt component (Beta-catenin). **(B)** Analysis of 26-week old mice.

To complement the qPCR-based experiments, we analyzed the expression of downstream effectors of Kras, Hedgehog, and Wnt signaling by immunostaining (Figure 3). To analyze the activity of Kras effector pathways, we performed immunostaining for AKT and MAPK signaling components (ERK). Immunostaining of the activated form of ERK and AKT (phospho-ERK1/2 and phospho-AKT) at 15 and 26 weeks revealed elevated p-ERK and p-AKT in PanINs, independent of genotype. While we did not observe changes in Kras effector pathways, the progressive loss of Notch inhibition in our model does not allow a rigorous epistatic analysis of these pathways.

To examine whether Notch inhibition in the tumor epithelium affected the expression of Hedgehog ligands, we performed immunostaining against Sonic Hedgehog (Shh). Shh is commonly upregulated in PanIN lesions [5, 32, 33, 36]. Studies have shown that upregulation of Hedgehog signaling promotes tumorigenesis [5, 32, 33, 36]. We detected Shh in the PanIN epithelium and not in the surrounding stroma, in both KC and KC-DNMAML; thus the expression of the Hedgehog pathway ligand Shh was not regulated by Notch signaling. To assess Wnt signaling activity *in vivo,* we performed immunostaining against beta-catenin. We did not observe changes in expression or subcellular localization of beta-catenin in the PanIN epithelium comparing KC and KC-DNMAML mice.

### Determining the requirement of Notch signaling during pancreatitis-driven PanIN formation

**Figure 4:**
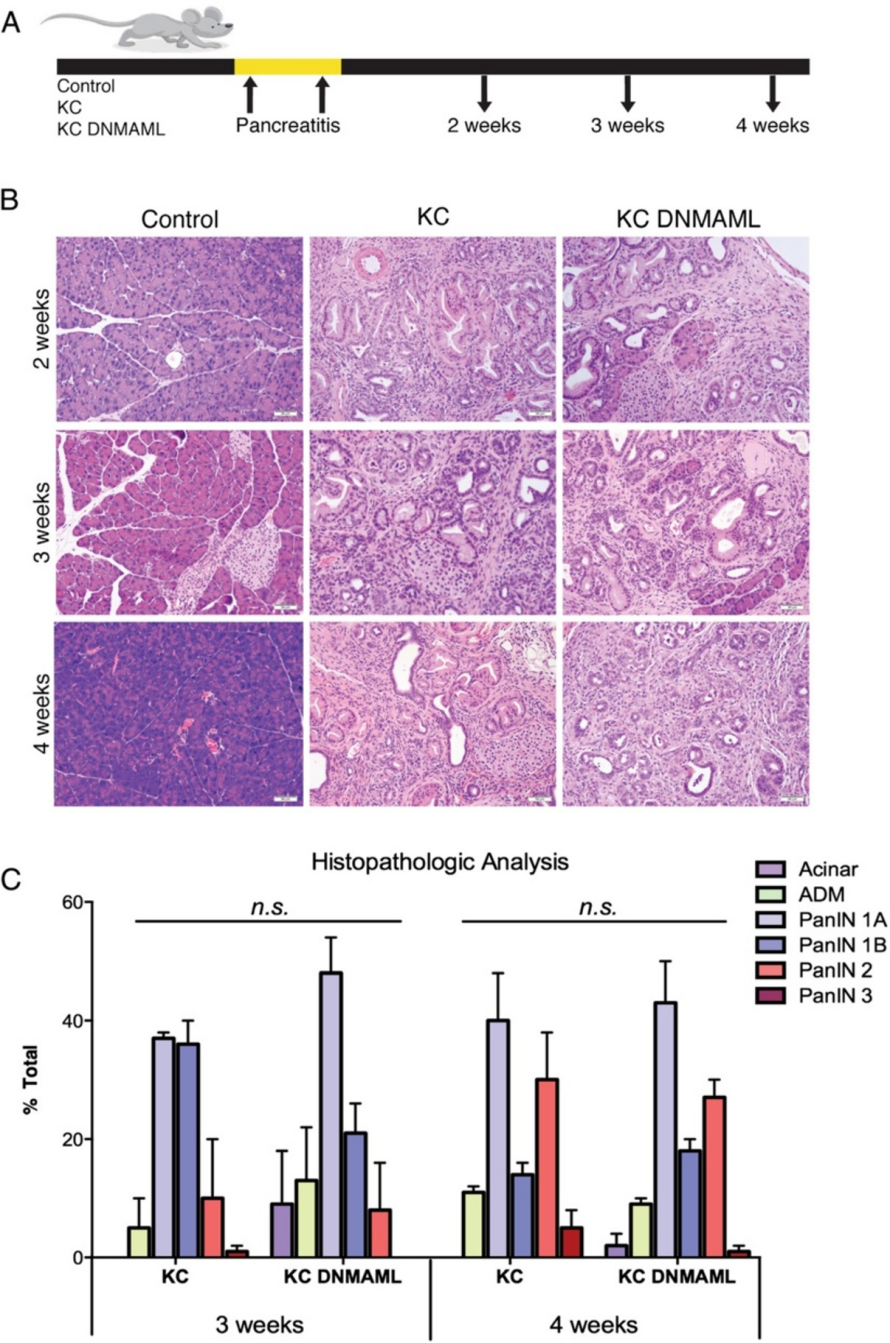
**Effect of Notch inhibition during pancreatitis-induced PanIN formation – Preliminary data. (A)** Age-matched wild type (control), *p48Cre;LSL-Kras* ^*G12D*^ (KC), and *p48Cre;LSL-Kras* ^*G12D*^ *; Rosa* ^*DNMAML-GFP/+*^ (KC;DNMAML) mice were administered intraperitoneal injections of caerulein, a cholecystokinin agonist, over the course of 48 hours. Pancreata was harvested and analyzed for PanIN development. (*n* = 2 mice/time point). **(B)** H&E staining of caerulein-treated wild type (control), *p48Cre;LSL-Kras* ^*G12D*^ (KC), and *p48Cre;LSL-Kras* ^*G12D*^ *; Rosa* ^*DNMAML-GFP/+*^ (KC;DNMAML) pancreata at high magnification (20x). (*n* = 2 mice/time point). **(C)** Quantification of pancreatic intraepithelial neoplasia (PanIN) at the indicated time points of age-matched *p48Cre;LSL-Kras* ^*G12D*^ (KC), and *p48Cre;LSL-Kras* ^*G12D*^ *; Rosa* ^*DNMAML-GFP/+*^ (KC;DNMAML) mice (*n* = 2 mice/genotype). Color coding as follows: acini (dark purple), acinar-ductal metaplasia (ADM; green), PanIN1A (light blue), PanIN1B (blue), PanIN2 (red), PanIN3 (dark red). Data represents mean ± SEM. *n.s*.: not statistically significant.

In our previous set of experiments, KC and KC;DNMAML mice developed PanINs spontaneously, over time. Several studies have reported that, in mice, the induction of acute pancreatitis synergizes with oncogenic Kras to induce rapid and extensive PanIN formation [6, 24, 37–39]. To investigate the potential requirement for active Notch signaling during pancreatitis-induced carcinogenesis, we administered a cholecystokinin (CCK) agonist (caerulein), which induces acute pancreatitis in mice (8 injections/day; 2 day treatment). We collected tissues from age-matched wild type (control), KC, and KC;DNMAML mice 2, 3, and 4 weeks following acute pancreatitis (Figure 4A). Acute pancreatitis leads to acinar damage, mainly represented by ADM, edema of the tissue, and infiltration of inflammatory cells both in WT and KC mice. However, while WT mice rapidly recover, with complete tissue repair usually observed within a week, KC mice are unable to undergo tissue repair (Figure 4B). In contrast, in KC mice, the pancreas becomes progressively fibrotic and the ADM becomes more extensive over the course of the first week after treatment. Then, the pancreas forms tissue-wide PanINs. The WT and KC cohorts in our experiment behaved as expected.. Similarly, KC;DNMAML developed PanINs, although they retained more acinar clusters 2 and 3 weeks after pancreatitis, compared to KC animals (Figure 4B,C). However, by 4 weeks after pancreatitis the two cohorts were indistinguishable. Based on our results from mice aging in absence of pancreatitis, this result might reflect progressive loss of Notch inhibition paralleling PanIN formation.

## Discussion

The Notch signaling pathway plays an important role in embryonic development of the pancreas, in homeostasis of the adult pancreatic tissues, and it is reactivated during pancreatic cancer [4, 5, 17, 40–42]. Several studies showed activation of Notch signaling as an early event in the development of pancreatic cancer [9–12], making inhibition of Notch signaling an attractive therapeutic target. Inhibition of Notch signaling in all cellular compartments by GSI treatment revealed that Notch is important for the initial development of PanINs and their progression to advanced PDA in mice [10]. A caveat to using GSIs is that they not only inhibit Notch signaling within the epithelium, but also in the tumor microenvironment (i.e. stellate cells, immune cells, fibroblasts). Thus, the ability of GSIs to globally inhibit Notch signaling, regardless of cell type, creates a challenge when teasing apart its role in epithelial cells. Mouse models that conditionally ablate Notch receptors in the pancreas epithelium demonstrated that Notch2, but not Notch3, is critical for PanIN initiation [16], although Notch3 is also upregulated during PanIN/PDA development [9, 10]. A limitation of genetic inactivation of individual Notch receptors is that they have been shown to functionally compensate for each other [43]. Thus, none of these previous studies has addressed the role of epithelia-specific Notch signaling mediated by any of the Notch receptors.

In this study, we used an approach that allowed conditional, tissue-specific inhibition of canonical Notch signaling specifically in the pancreas epithelium (in a cell autonomous manner). Using a dominant negative form of Mastermind-like1 (DNMAML), we successfully inhibited transcriptional activation of Notch target genes, independent of Notch receptor input [21, 22], in the pancreatic epithelium. DNMAML expression was previously shown to lead to impaired endocrine cell differentiation [44, 45]. While a comprehensive characterization of the endocrine cells went beyond the scope of the current study, the mice presented with morphologically normal islets, probably reflecting differences in transgene expression levels or timing of expression. Our results showed delay, but not blockade, of PanIN formation. In part, this finding might be due to an escape mechanism allowing Notch signaling to be reactivated even in DNMAML expressing mice – either through transgene silencing, or possibly though sequestration of DNMAML away from the nucleus. Thus, epithelial Notch signaling appears to be required for the onset of PanIN formation. However, selection mechanisms lead to accumulation of cells with active Notch signaling. Our data do not address the potential role of Notch signaling in other cellular compartments, such as fibroblasts or the immune system. However, an initial analysis revealed little presence of Notch target components in fibroblasts. The status and potential role of Notch signaling in immune cells at different stages of cancer formation cannot be discounted and should be addressed in future studies.

## Conclusion

Notch signaling is important for disease initiation early in PanIN development. However later in carcinogenesis, the role of Notch signaling in the tumor epithelium remains unclear. Future studies examining the role of Notch signaling in other cells within the tumor microenvironment (immune cells) may explain a purpose for maintaining Notch signaling throughout carcinogenesis. Notch inhibition is still an attractive target for neo-adjuvant or prophylactic treatment in high-risk patients, but more work is needed.

## Electronic supplementary material

Additional file 1: Table S1: Antibodies. **Table S2.** Primer sequences for quantitative RT-PCR. (DOCX 87 KB)

## Abbreviations

PanIN: Pancreatic intraepithelial neoplasia
GSI: Gamma secretase inhibitor
MAML: Mastermind-like
DNMAML: Dominant-negative form of Mastermind-like
ADM: Acinar-ductal metaplasia
PDA: Pancreatic ductal adenocarcinoma
NICD: Notch intracellular domain.
