# Supplementary material for: Epithelial Notch signaling is a limiting step for pancreatic carcinogenesis
Source: BMC Cancer. 2014 Nov 22;14:862. doi: 10.1186/1471-2407-14-862 (PMC4289235; doi:10.1186/1471-2407-14-862)
Supplement: Supplementary file 1 — Additional file 1: Table S1: Antibodies. Table S2. Primer sequences for quantitative RT-PCR. (DOCX 87 KB) [file 12885_2014_5115_MOESM1_ESM.docx]

**SUPPLEMENTAL MATERIAL**

**Table S1:** Antibodies

| Antibody | Vendor | Catalog Number | Dilution |
| --- | --- | --- | --- |
| Primary Antibody |  |  |  |
| Beta-catenin | Cell Signaling | 9587 | 1:100 |
| Claudin18 | Life Technologies | 700178 | 1:150 |
| GFP | Abcam | Ab13970 | 1:2000 |
| Hes1 | Ben Stanger | - | 1:1500 |
| Ki67 | Vector Laboratories | VP-RM04 | 1:100 |
| p-AKT | Cell Signaling | 9271 | 1:100 |
| p-ERK ½  (phosphor-p44/42) | Cell Signaling | 4370 | 1:100 |
| Shh | R&D Systems | AF445 | 1:100 |

Table S2: Primer sequences for quantitative RT-PCR

| Gene | Forward Primer | Reverse Primer |
| --- | --- | --- |
| *Hes1* | CCAGCCAGTGTCAACACGA | AATGCCGGGAGCTATCTTTCT |
| *Hey1* | GCGCGGACGAGAATGGAAA | TCAGGTGATCCACAGTCATCTG |
| *Hey2* | AAGCGCCCTTGTGAGGAAAC | GGTAGTTGTCGGTGAATTGGAC |
| *Ptch1* | TTGTGGAAGCCACAGAAAACC | TGTCTGGAGTCCGGATGGA |
| *Wnt3a* | GCACCACCGTCAGCAACA | GGGTGGCTTTGTCCAGAACA |
